# Identification of cuproptosis-related gene SLC31A1 and upstream LncRNA-miRNA regulatory axis in breast cancer

**DOI:** 10.1038/s41598-023-45761-5

**Published:** 2023-10-26

**Authors:** Jia-hao Wu, Tian-cheng Cheng, Bei Zhu, Hai-yan Gao, Lin Zheng, Wei-xian Chen

**Affiliations:** 1 Department of Breast Surgery, The Affiliated Changzhou No.2 People’s Hospital of Nanjing Medical University, 29 Xinglongxiang, Changzhou, 213000 Jiangsu Province China; 2 Graduate School, Dalian Medical University, Dalian, 116000 Liaoning Province China; 3 Graduate School, Bengbu Medical College, Bengbu, 233000 Anhui Province China; 4 Department of Breast Surgery, The Affiliated Changzhou Tumor Hospital of Soochow University, Changzhou, 213000 Jiangsu Province China; 5 Post-doctoral Working Station, The Affiliated Changzhou No.2 People’s Hospital of Nanjing Medical University, Changzhou, 213000 Jiangsu Province China

**Keywords:** Breast cancer, Tumour biomarkers

## Abstract

Mounting evidence indicate that cuproptosis, a novel form of programmed cell death, contributes to cancer development and progression. However, a comprehensive analysis regarding the expressions, functions, and regulatory network of cuproptosis-related genes is still lacking. In the present work, cuproptosis-related genes, upstream miRNAs and lncRNAs, and clinical data of breast cancer from TCGA database were analyzed by R language including Cox regression analysis, correlation calculation, ROC curve construction, and survival evaluation, and were further verified by public-available databases. Chemosensitivity and immune infiltration were also evaluated by online tools. SLC31A1 was significantly increased in breast cancer samples than those in normal tissues. SLC31A1 was negatively related to a favorable outcome in breast cancer, and the AUC value increased with the prolongation of follow-up time. LINC01614 and miR-204-5p were potential upstream regulators of SLC31A1. Moreover, SLC31A1 was significantly positively correlated with different immune cells infiltration, immune cell biomarkers, and immune checkpoints in breast cancer. SLC31A1 was a potential cuproptosis-related gene in breast cancer, which was significantly upregulated and was able to predict diagnosis, prognosis, chemosensitivity, and immune infiltration. LINC01640/miR-204-5p/SLC31A1 might be a significant and promising axis during cuproptosis in breast cancer.

## Introduction

Breast cancer is one of the leading causes of death among females^1^. Though great progress has been made in screening, diagnosis, and treatment of breast cancer during the past few decades, the overall survival remains poor in breast cancer patients^2^. Therefore, it is significant to investigate molecular mechanisms of breast cancer and discover new targets and therapeutic strategies to improve clinical outcomes.

Copper is one of the fundamental materials in various biological processes including mitochondrial respiration^3^. A recent publication revealed that intracellular copper accumulation contributed to the aggregation of mitochondrial lipoylated proteins, leading to a novel form of programmed cell death termed cuproptosis^3^. Accumulating evidence suggest that dysregulated copper level is responsible for oncogenesis and cancer progression^4^. Unlike oxidative stress-related cell death such as apoptosis, ferroptosis, and necroptosis, studies of cuproptosis in cancer prognosis and immunity are still lacking.

Recently, several copper metabolism genes, including FDX1, SLC31A1, ATP7B, and CDKN2, have been reported^5–8^. Researchers have confirmed that aberrant regulation of these cuproptosis genes were closely related to cancer development, immune response evaluation, and prognosis prediction^4,9–11^. To date, the expressions, functions, prognostic values, and regulatory network of cuproptosis-related genes and their correlation with immune landscape in breast cancer have not been fully elucidated. In the present work, several cuproptosis-related genes were collected. Using R language and multiple network databases, expression levels, prognostic and diagnostic values of cuproptosis-related gene SLC31A1 were analyzed in breast cancer. Potential upstream miRNAs and lncRNAs of SLC31A1, significance of SLC31A1 in chemosensitivity and immune response were also evaluated. This study might provide important information to uncover the role of cuproptosis-related gene SLC31A1 in breast cancer and might offer a new strategy to treat breast cancer through intervening the LINC01614/miR-204-5p/SLC31A1 axis.

## Materials and methods

### Data download and analysis

TCGA (https://www.cancer.gov/about-nci/organization/ccg/research/structural-genomics/tcga) generates over 2.5 petabytes of genomic, epigenomic, transcriptomic, and proteomic data. These data are publicly available for anyone to download and analyze during cancer research. R is a freely downloadable language and environment for statistical computing and graphics^12^. R provides a wide variety of statistical and graphical techniques, and is highly extensible. In the present study, a total of 1097 clinical data, 1027 miRNAs, 1226 mRNAs and 1226 lncRNAs were downloaded from TCGA database, patients with no prognostic information or incomplete gene expression data were excluded to ensure accurate measurement. Differential analysis, univariate Cox analysis, and correlation analysis were also performed by using the limma, pheatmap, survival, and timeROC packages in R language.

### starBase analysis

starBase (http://starBase.sysu.edu.cn/), an online tool updated to provide the most comprehensive analysis of miRNA-mRNA and miRNA-lncRNA interaction networks from large-scale CLIP-Seq data, was used to evaluate the expression levels of cuproptosis-related genes, miRNAs and lncRNAs^13^. In the present work, connection of SLC31A1-miRNA, SLC31A1-lncRNA, miRNA-lncRNA, and SLC31A1-immune landscape in breast cancer was accessed by starBase.

### GEPIA analysis

GEPIA (http://gepia.cancer-pku.cn/) is a web server to profile gene expressions between tumor tissues and normal samples from the TCGA and GTEx projects and to explore correlations between gene expressions and patients survival^14^. In the present study, expression levels and prognostic values of cuproptosis-related genes in breast cancer were analyzed by GEPIA. Correlations among SLC31A1, immune genes, and immune cells biomarkers in breast cancer were also confirmed by using GEPIA database.

### Kaplan–Meier plotter analysis

Kaplan–Meier plotter (http://kmplot.com/analysis/) is a web-based platform for evaluating the relevance between gene expressions and survival^15^. In the present work, Kaplan–Meier plotter was employed to analyze the clinical prognostic significance of cuproptosis-related genes, miRNAs, and lncRNAs in breast cancer patients, including overall survival.

### PrognoScan analysis

PrognoScan (http://dna00.bio.kyutech.ac.jp/PrognoScan/) is a large database for assessing the biological relationship between gene expressions and prognostic information including relapse free survival (RFS) and distant metastasis free survival (DMFS) in breast cancer patients across a large collection of publicly available cancer microarray datasets^16^.

### GSCA analysis

GSCA (http://bioinfo.life.hust.edu.cn/GSCA/#/) is an integrated platform for cancer analysis at genomic, pharmacogenomic and immunogenomic levels^17^. In the present study, GSCA was used to analyze cuproptosis-related genes expressions, tumor immune infiltration, drug sensitivity, and their associations with clinical outcomes.

### TIMER analysis

TIMER (http://cistrome.shinyapps.io/timer/) is a web server for systematic analysis of tumor-infiltrating immune cells across multiple cancer types^18^. In the present work, TIMER was employed to comprehensively evaluate the correlation of SLC31A1 with immune cells markers, tumor-infiltrating immune cells and immune check points in breast cancer.

### CancerMIRNome

CancerMIRNome (http://bioinfo.jialab-ucr.org/Cancer-MIRNome) is an online resource for cancer miRNome interactive analysis and visualization based on the human miRNome data from 33 cancer types in TCGA^19^. In the present study, CancerMIRNome was applied to plot ROC curves and survival analysis, by using the upstream miRNAs of cuproptosis-related genes SLC31A1 filtered by R.

### CTR-DB analysis

CTR-DB (http://ctrdb.ncpsb.org.cn/) is a database designed for researchers to collect and evaluate patient-derived clinical transcriptomes with cancer drug response^20^. In the present work, CTR-DB was employed to discover the role of SLC31A1 in predicting chemotherapeutic sensitivity in breast cancer.

### PPI network and enrichment analysis

Protein–protein interaction (PPI) information and networks for SLC31A1 gene were analyzed by using the Search Tool for the Retrieval of Interacting Genes (STRING) database (http://www.string-db.org/). Then, pathway enrichment analysis was performed. Cytoscape (http://cytoscape.org/) was used to construct the possible functional network.

### PCR validation

A total of 4 pairs of breast cancer samples and normal breast tissues were collected from the Affiliated Changzhou No.2 People’s Hospital of Nanjing Medical University. Collection and analysis of clinical samples were conducted according to the Declaration of Helsinki and approved by the ethics committee of Changzhou No.2 People’s Hospital. Informed written consent was received from all patients. Briefly, total RNA was obtained by using the TRIzol reagent (Thermo Fisher Scientific, USA), and then transcribed into cDNA by using the Hiscript II qRT SuperMix (Vazyme, China). Quantitative real‐time PCR was carried out by using the AceQ qPCR SYBR Green Master Mix (Vazyme, China) on a ViiA 7 Real-Time PCR System (Thermo Fisher Scientific, USA). Primer sequences used for PCR are shown in Supplementary Table 1. All reactions, including the negative controls, were performed in triplicate.

### Statistical analysis

All the results in this study were automatically analyzed by the above-mentioned online database or R language. P-values < 0.05 or logrank P-value < 0.05 was defined as significant difference.

### Ethics statement

The ethics committee of the Affiliated Changzhou No.2 People’s Hospital of Nanjing Medical University approved the present study. Collection and analysis of clinical samples were conducted in accordance with the Declaration of Helsinki and approved by the ethics committee of Changzhou No.2 People’s Hospital. Informed written consent was received from all patients.

## Results

### Expressions of cuproptosis-related genes in breast cancer

**Figure 1:**
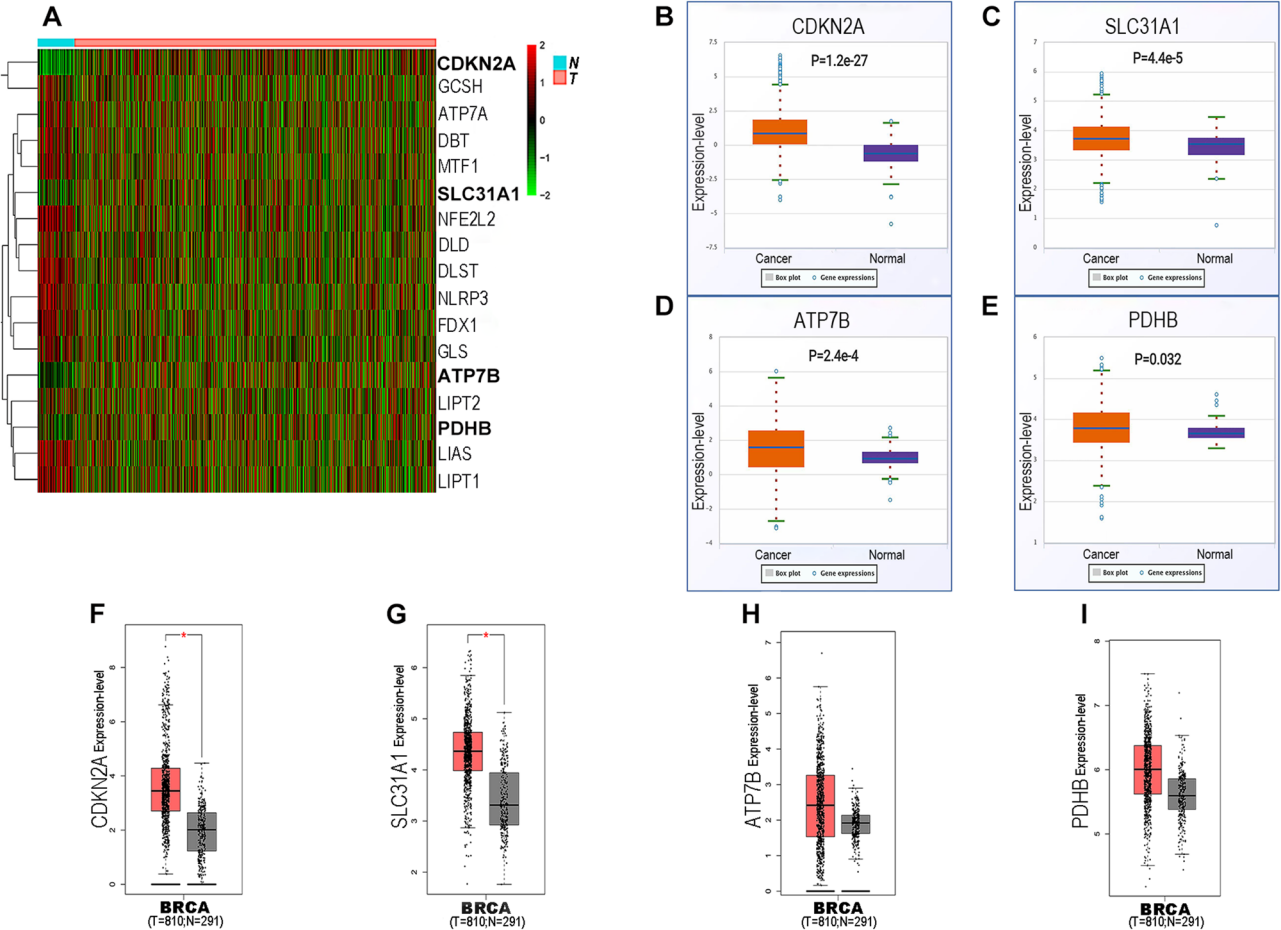
Expressions of cuproptosis-related genes in breast cancer. (**A**) Heat map showing differentially expressed cuproptosis-related genes in breast cancer was analyzed by the limma package and pheatmap package in R language. Expression levels of CDKN2A (**B**), SLC31A1 (**C**), ATP7B (**D**), and PDHB (**E**) in breast cancer were analyzed by starBase. Expression levels of CDKN2A (**F**), SLC31A1 (**G**), ATP7B (**H**), and PDHB (**I**) in breast cancer were analyzed by GEPIA.

In the present work, 1097 clinical data, 1027 miRNAs, 1226 mRNAs, and 1226 lncRNAs were downloaded from TCGA database. Patients with no prognostic information or incomplete gene expression data were excluded to ensure accurate measurement. In all 1076 female breast cancer patients with the mean age of 58.1 ± 12.9 ranging from 26 to 90, there were 16.9% stage I, 55.8% stage II, 24.3% stage III, and 3% stage IV. By reviewing the publications, a number of cuproptosis-related genes were enrolled for evaluation, consisting of ATP7A, ATP7B, CDKN2A, DBT, DLAT, DLD, DLST, FDX1, GCSH, GLS, LIAS, LIPT1, LIPT2, MTF1, NFE2L2, NLRP3, PDHA1, PDHB, and SLC31A1^21–23^. A total of 17 cuproptosis-related genes were differentially expressed between breast cancer samples and normal tissues by using the limma package and pheatmap package in R language (Fig. 1A). Specifically, NFE2L2, NLRP3, ATP7A, FDX1, LIAS, LIPT1, LIPT2, DLD, MTF1, GLS, DBT, GCSH, and DLST were higher expressed in normal tissues compared with tumor samples, while CDKN2A, SLC31A1, ATP7B, and PDHB were higher expressed in breast cancer tissues (Table 1). Consistent with the data from R language, starBase showed that expression levels of CDKN2A, SLC31A1, ATP7B, and PDHB were markedly up-regulated in breast cancer with respect to normal samples (Fig. 1B–E). Subsequently, GEPIA was also employed to confirm the expressions of cuproptosis-related genes in breast cancer. The results showed that only CDKN2A and SLC31A1 were significantly increased in breast cancer samples than those in normal tissues (Fig. 1F–I). Thus, CDKN2A and SLC31A1 may be two most potential genes associated with cuproptosis in breast cancer.

**Table 1:** Expressions of cuproptosis-related genes in breast cancer analyzed by R.

Gene	logFC	P-value
ATP7A	− 0.046924832	0.000211129***
**ATP7B**	**0.072818761**	5.13E−06***
**CDKN2A**	**0.320006**	3.40E−30***
DBT	− 0.038437545	3.71E−05***
DLD	− 0.031446601	0.000419659***
DLST	− 0.055429552	3.98E−17***
FDX1	− 0.057734168	1.92E−10***
GCSH	− 0.053088522	0.002499222**
GLS	− 0.042434435	1.33E−06***
LIAS	− 0.085838335	3.38E−16***
LIPT1	− 0.091340578	9.25E−16***
LIPT2	− 0.030624884	0.043870075*
MTF1	− 0.043579927	9.23E−06***
NFE2L2	− 0.060453683	7.24E−16***
NLRP3	− 0.152620853	5.00E−14***
**PDHB**	**0.011965182**	0.012411176*
**SLC31A1**	**0.031825173**	9.12E−05***

*P-value < 0.05; **P-value < 0.01; ***P-value < 0.001; values in bold indicate that these cuproptosis-related genes are upregulated in breast cancer and these results are statistically significant.

### Survival analysis of cuproptosis-related genes in breast cancer

**Figure 2:**
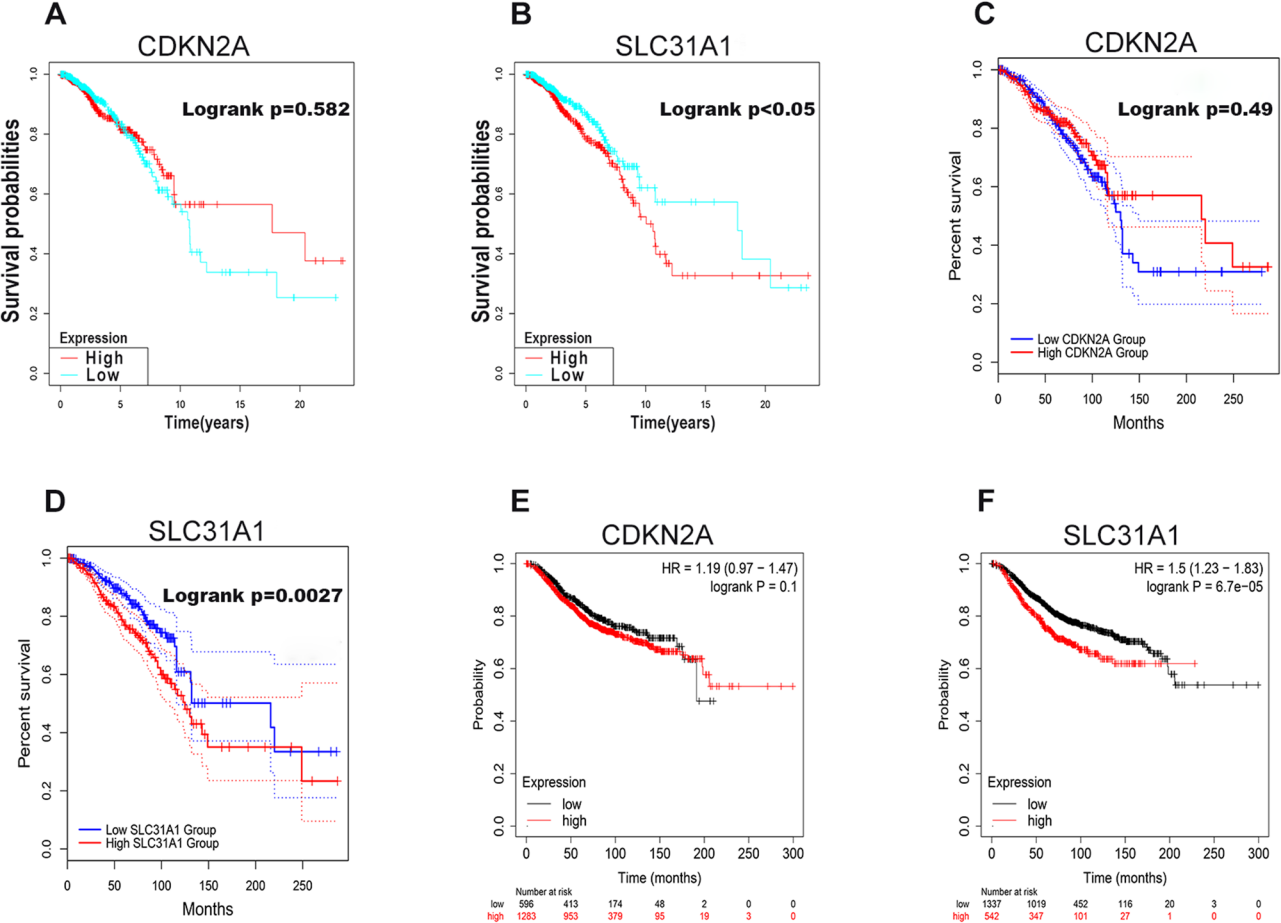
Overall survival analysis of CDKN2A and SLC31A1 in breast cancer. Prognostic values of CDKN2A (**A**) and SLC31A1 (**B**) in breast cancer were analyzed by the survival package in R language. Prognostic values of CDKN2A (**C**) and SLC31A1 (**D**) in breast cancer were accessed by GEPIA. Prognostic values of CDKN2A (**E**) and SLC31A1 (**F**) in breast cancer were measured by Kaplan–Meier plotter.

**Figure 3:**
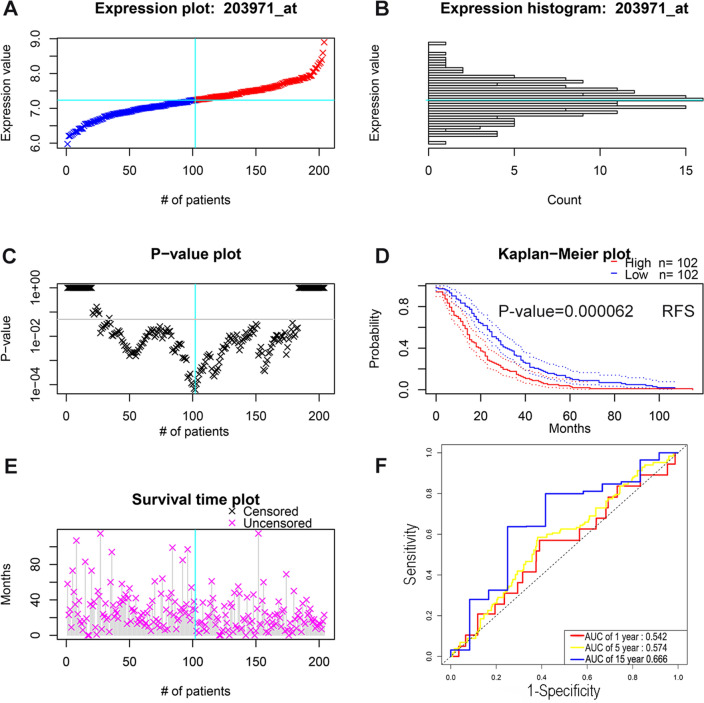
Prognostic value of SLC31A1 in breast cancer verified using GSE12276 dataset. Expression plot (**A**), expression histogram (**B**), P-value plot (**C**), Kaplan–Meier plot for relapse free survival (**D**), survival time plot (**E**), and ROC curve of 1, 5, and 15 years of SLC31A1 (**F**) in breast cancer patients of GSE12276 dataset were analyzed by PrognoScan.

**Figure 4:**
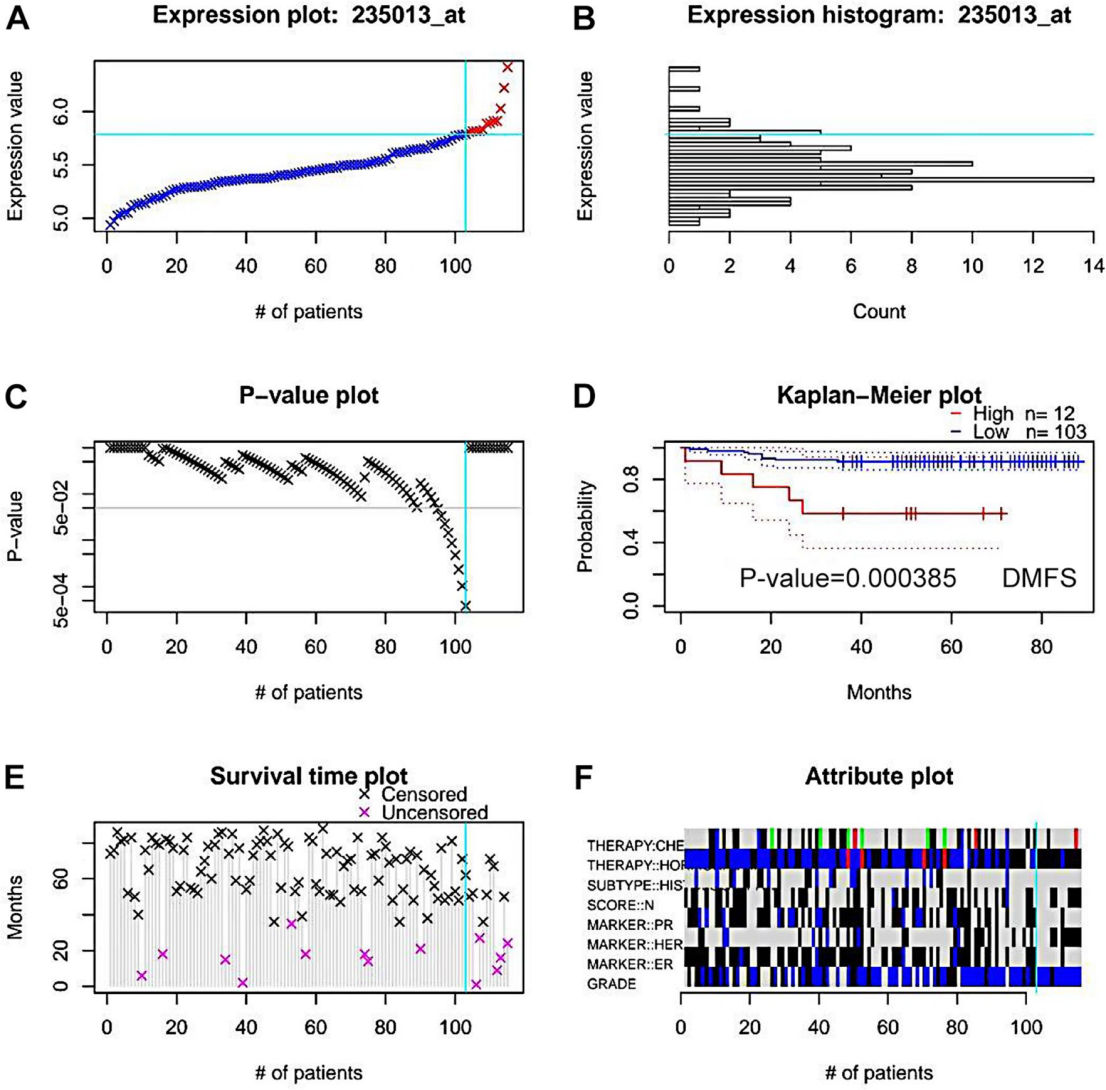
Prognostic value of SLC31A1 in breast cancer verified using GSE19615 dataset. Expression plot (**A**), expression histogram (**B**), P-value plot (**C**), Kaplan–Meier plot for distant metastasis free survival (**D**), survival time plot (**E**), and attribute plot (**F**) of SLC31A1 in breast cancer patients of GSE19615 dataset were analyzed by PrognoScan.

Prognostic values of two cuproptosis-related genes in breast cancer, namely CDKN2A and SLC31A1, were then evaluated. First, overall survival analysis was performed by using the survival package in R language. In particular, no statistical prognostic value of CDKN2A in breast cancer was observed (Fig. 2A), while patients with higher expression of SLC31A1 displayed a poor prognosis (Fig. 2B). GEPIA was utilized to carry out overall survival analysis. For CDKN2A, no statistical difference of overall survival was found between lower group and higher group in breast cancer (Fig. 2C). However, group with lower expression of SLC31A1 exhibited a better overall survival, with respect to group with higher expression of SLC31A1 (Fig. 2D). Kaplan–Meier plotter was also applied for verification. Only SLC31A1, but not CDKN2A, was negatively related to a favorable overall survival in breast cancer (Fig. 2E, F). Therefore, SLC31A1 was chosen for further investigation. Two GEO datasets, GSE12276 and GSE19615, were introduced to verify the predictive value of SLC31A1 in prognosis by using PrognoScan. The results showed that breast cancer patients with high expression of SLC31A1 had a poor relapse free survival (Fig. 3A–E) and distant metastasis free survival (Fig. 4A–F). Taken together, these findings suggest that SLC31A1 might be the most potential cuproptosis-related gene and could be a promising unfavorable prognostic biomarker in breast cancer.

### Expression, PPI network, enrichment analysis, and ROC curve of SLC31A1

Gene expression of SLC31A1 in breast cancer samples and adjacent normal tissues were analyzed. Expression level of SLC31A1 was increased in breast cancer samples with respect to normal tissues (Supplementary Fig. 1A). Protein–protein interaction information and networks (Supplementary Fig. 1B) for SLC31A1 gene were analyzed by STRING database and Cytoscape software, including CCS, SLC11A2, ATP7A, ATP7B, ATOX 1, SLC22A2, SLC22A1, SLC30A1, CP, and COX 17. Enrichment pathway analysis showed that the genes were related to copper ion binding and transport (Supplementary Fig. 1C, D). In order to further evaluate the diagnostic value of cuproptosis-related gene SLC31A1 in breast cancer, ROC curve of SLC31A1 was drawn by using the survival package and timeROC package in R language based on breast cancer samples and normal breast tissues from TCGA. According to the ROC curve, SLC31A1 displayed the ability to distinguish breast cancer samples from normal breast tissues, and the AUC value increased with the prolongation of follow-up time (Fig. 3F). Specifically, AUC of 1 year, 5 year, and 15 year was 0.542, 0.574, and 0.666, indicating that SLC31A1 might also be a promising diagnostic marker in breast cancer.

### Prediction of upstream miRNAs of SLC31A1 in breast cancer

**Figure 5:**
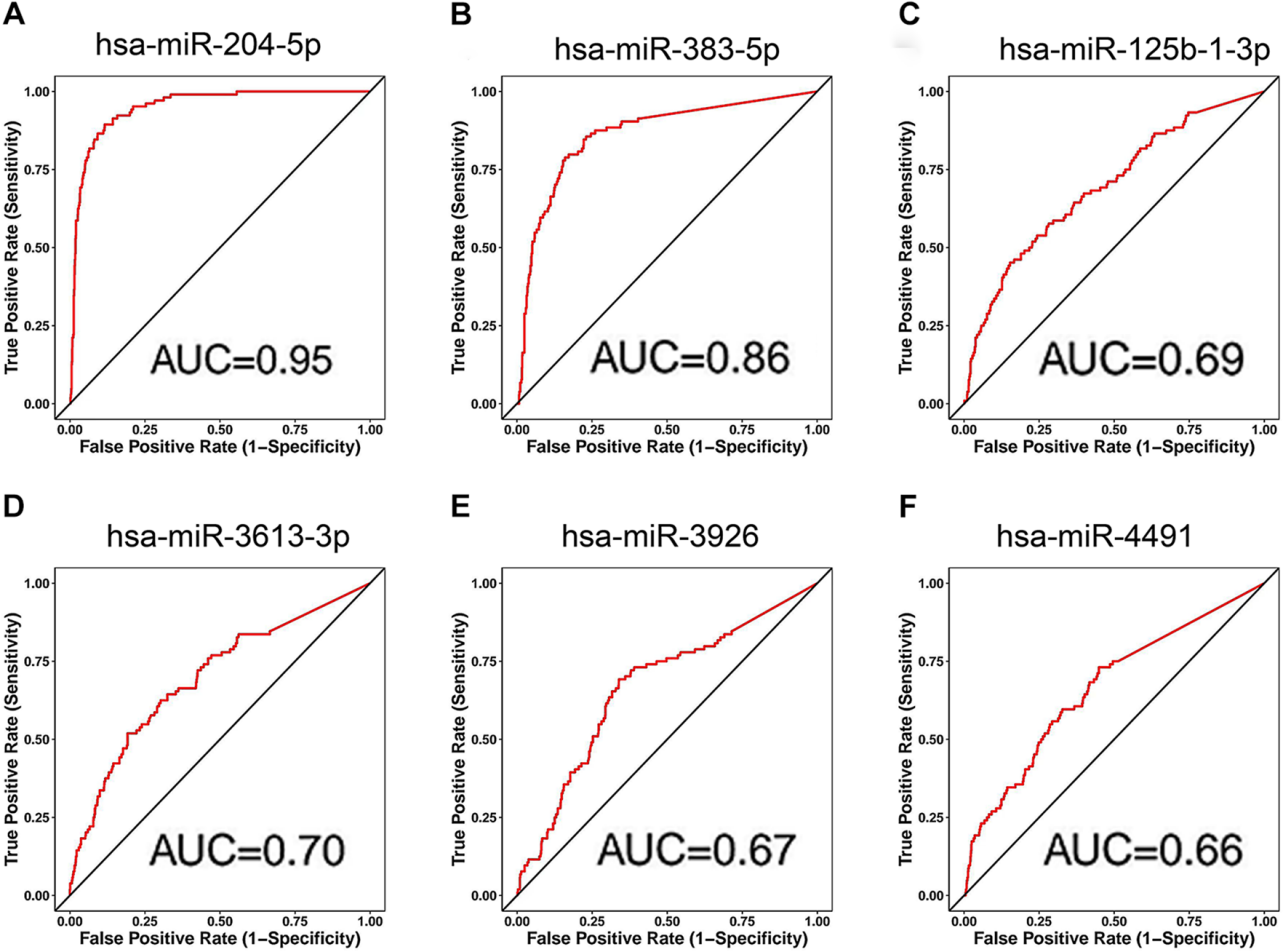
Diagnostic values for miRNAs in breast cancer. Diagnostic values of miR-204-5p (**A**), miR-383-5p (**B**), miR-125b-1-3p (**C**), miR-3613-3p (**D**), miR-3926 (**E**), and miR-4491 (**F**) in breast cancer were assessed by CancerMIRNome.

**Figure 6:**
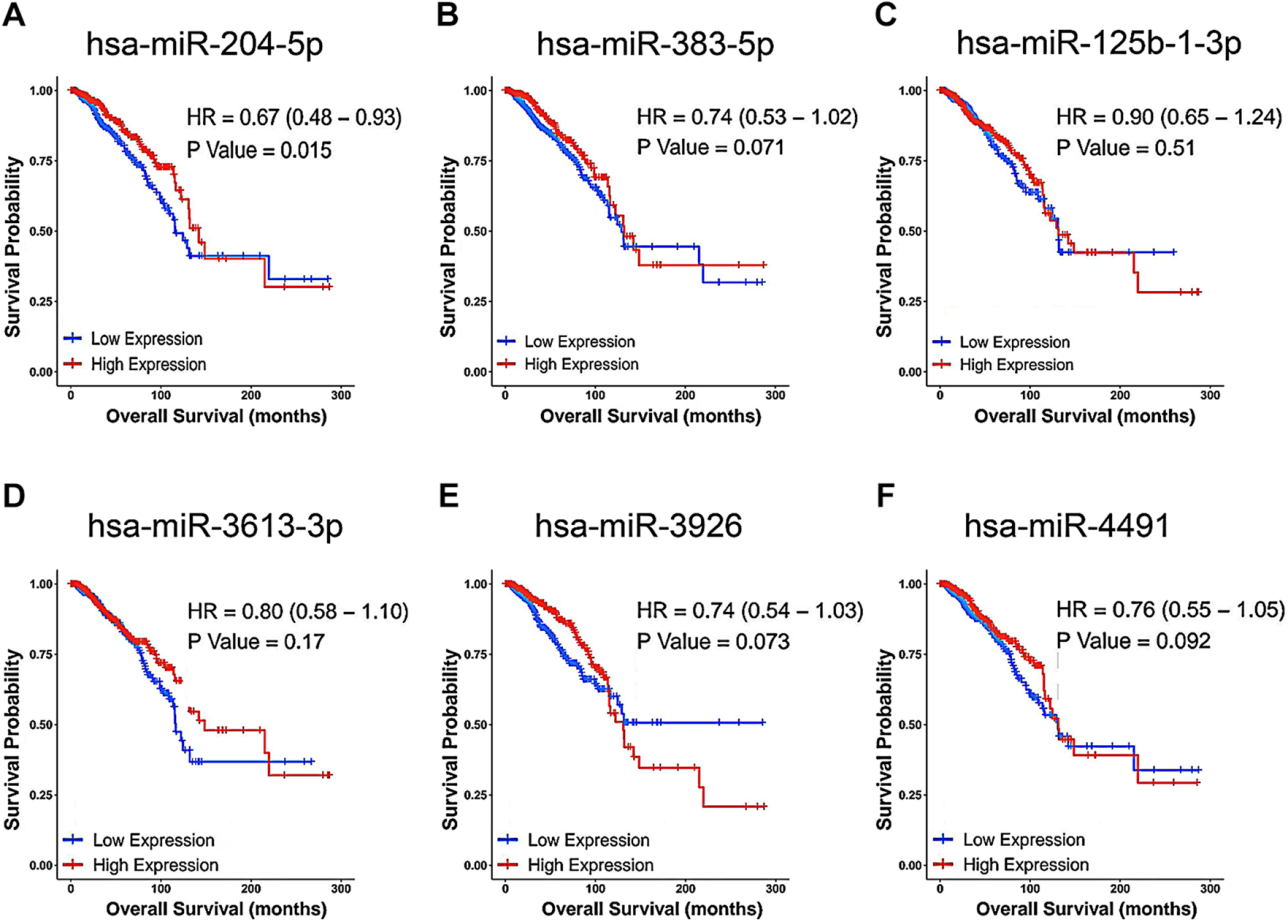
Prognostic values for miRNAs in breast cancer. Prognostic values of miR-204-5p (**A**), miR-383-5p (**B**), miR-125b-1-3p (**C**), miR-3613-3p (**D**), miR-3926 (**E**), and miR-4491 (**F**) in breast cancer were evaluated by CancerMIRNome.

As known to all, miRNAs are a class of single-stranded and noncoding RNA that serve as posttranscriptional regulators of gene expression^24^. To determine whether cuproptosis-related gene SLC31A1 could be modulated by corresponding miRNAs, upstream miRNAs of SLC31A1 were predicted. First, differentially expressed miRNAs of breast cancer were comprehensively profiled through the limma package in R language, and univariate Cox regression analysis was performed on the obtained 191 down-regulated miRNAs. A total of 8 miRNAs were enrolled, followed by Pearson correlation calculation between SLC31A1 and 8 miRNAs using the limma package in R language. Only 6 miRNAs, namely miR-125b-1-3p, miR-204-5p, miR-3613-3p, miR-383-5p, miR-3926, and miR-4491, were negatively correlated (Table 2). Then, diagnostic and prognostic values of 6 miRNAs in breast cancer were accessed by CancerMIRNome. As shown in Fig. 5, miR-204-5p (AUC = 0.95), miR-383-5p (AUC = 0.86), miR-125b-1-3p (AUC = 0.69), miR-3613-3p (AUC = 0.70), miR-3926 (AUC = 0.67), and miR-4491 (AUC = 0.66) displayed potential diagnostic values in breast cancer. Among the 6 miRNAs, only breast cancer patients with higher expression of miR-204-5p had a better overall survival according to the results from prognosis analysis (Fig. 6). It is therefore concluded that miR-204-5p might be an upstream miRNA of cuproptosis-related gene SLC31A1.

**Table 2:** Correlation between SLC31A1 and 8 predicted miRNAs in breast cancer analyzed by R.

miRNA	Correlation	P-value
miR-1258	− 0.02415	0.428755
**miR-125b-1-3p**	− **0.0847**	0.005435**
**miR-204-5p**	− **0.14236**	2.75E−06***
**miR-3613-3p**	− **0.10771**	0.000401***
**miR-383-5p**	− **0.13882**	4.87E−06***
**miR-3926**	− **0.09756**	0.001354**
**miR-4491**	− **0.08563**	0.004942**
miR-6766-3p	− 0.00037	0.990316

*P-value < 0.05; **P-value < 0.01; ***P-value < 0.001; values in bold indicate that these results are statistically significant.

### Prediction of upstream lncRNAs of miR-204-5p/SLC31A1 in breast cancer

**Figure 7:**
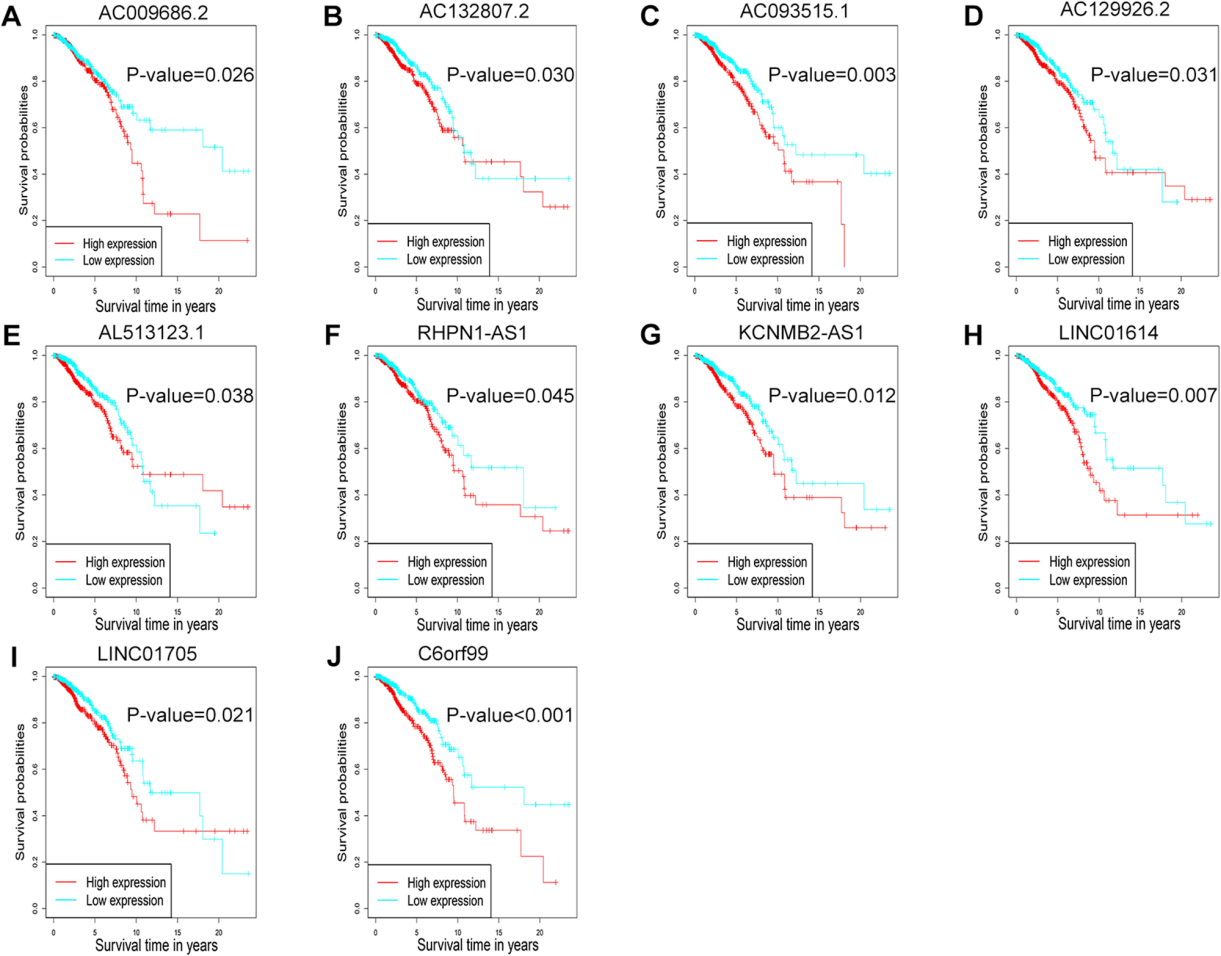
Prognostic values for lncRNAs in breast cancer. Prognostic values of AC009686.2 (**A**), AC132807.2 (**B**), AC093515.1 (**C**), AC129926.2 (**D**), AL513123.1 (**E**), RHPN1-AS1 (**F**), KCNMB2-AS1 (**G**), LINC01614 (**H**), LINC01705 (**I**), and C6orf99 (**J**) in breast cancer were assessed by the survival package in R language.

Potential upstream lncRNAs of the miR-204-5p/SLC31A1 axis were subsequently predicted. As described above, differentially expressed lncRNAs of breast cancer were comprehensively listed using the limma package in R language, and univariate Cox regression analysis was performed on the obtained 137 up-regulated lncRNAs. A total of 32 lncRNAs were enrolled, followed by Pearson correlation analysis between miR-204-5p and 32 lncRNAs (Table 3). Prognostic values were then analyzed on these 32 lncRNAs by the survival package in R language. The results demonstrated that breast cancer patients with increased levels of AC009686.2, AC132807.2, AC093515.1, AC129926.2, AL513123.1, RHPN1-AS1, KCNMB2-AS1, LINC01614, LINC01705, and C6orf99 had a better overall survival (Fig. 7A–J). Correlation analysis between SLC31A1 and the obtained 10 lncRNAs indicated that LINC01614 was most highly positively correlated with SLC31A1 (Table 4). Collectively, LINC01614/miR-204-5p/SLC31A1 might be a potential axis related to cuproptosis in breast cancer.

**Table 3:** Correlation between miR-204-5p and 32 predicted lncRNAs in breast cancer analyzed by R.

lncRNA	miRNA	R-value	P-value
LINC01929	hsa-miR-204-5p	− 0.19252	1.92E−10***
RHPN1-AS1	hsa-miR-204-5p	− 0.17395	9.30E−09***
C6orf99	hsa-miR-204-5p	− 0.14819	1.05E−06***
LINC01705	hsa-miR-204-5p	− 0.14134	3.25E−06***
WT1-AS	hsa-miR-204-5p	− 0.13314	1.18E−05***
LINC02201	hsa-miR-204-5p	− 0.11941	8.62E−05***
AP001434.1	hsa-miR-204-5p	− 0.11728	0.000115***
LINC01614	hsa-miR-204-5p	− 0.11573	0.000142***
AC129926.2	hsa-miR-204-5p	− 0.11098	0.000265***
LINC02257	hsa-miR-204-5p	− 0.10697	0.00044***
AC093515.1	hsa-miR-204-5p	− 0.10536	0.000537***
AL513123.1	hsa-miR-204-5p	− 0.09647	0.001535**
AC069061.2	hsa-miR-204-5p	− 0.0887	0.003591**
KCNMB2-AS1	hsa-miR-204-5p	− 0.08557	0.004971**
SIRLNT	hsa-miR-204-5p	− 0.08533	0.005099**
AC079160.1	hsa-miR-204-5p	− 0.08365	0.006042**
AC007128.2	hsa-miR-204-5p	− 0.08174	0.007302**
AC087591.1	hsa-miR-204-5p	− 0.07922	0.009333**
AC132807.2	hsa-miR-204-5p	− 0.07777	0.010713*
AC092448.1	hsa-miR-204-5p	− 0.07387	0.015374*
AC078983.1	hsa-miR-204-5p	− 0.0732	0.016323*
AC061975.6	hsa-miR-204-5p	− 0.07233	0.017648*
AC000067.1	hsa-miR-204-5p	− 0.0712	0.019508*
AC009686.2	hsa-miR-204-5p	− 0.07072	0.020345*
AC068189.1	hsa-miR-204-5p	− 0.07007	0.021523*
LINC01419	hsa-miR-204-5p	− 0.06961	0.022393*
LINC02726	hsa-miR-204-5p	− 0.06849	0.024659*
LINC02163	hsa-miR-204-5p	− 0.06847	0.024703*
AC117945.2	hsa-miR-204-5p	− 0.06551	0.031669*
LINC01615	hsa-miR-204-5p	− 0.06531	0.032186*
MIR3150BHG	hsa-miR-204-5p	− 0.0646	0.034105*
AL357033.2	hsa-miR-204-5p	− 0.0603	0.047969*

*P-value < 0.05; **P-value < 0.01; ***P-value < 0.001.

**Table 4:** Correlation between SLC31A1 and 10 predicted lncRNAs in breast cancer evaluated by R.

lncRNA	Gene	R-value	P-value
**LINC01614**	SLC31A1	**0.332329**	1.21E−29***
**RHPN1-AS1**	SLC31A1	**0.297741**	7.46E−24***
**C6orf99**	SLC31A1	**0.293081**	3.94E−23***
**AC093515.1**	SLC31A1	**0.184934**	7.01E−10***
**AC009686.2**	SLC31A1	**0.160801**	8.81E−08***
**LINC01705**	SLC31A1	**0.152798**	3.77E−07***
**AL513123.1**	SLC31A1	**0.106672**	0.000406***
**KCNMB2-AS1**	SLC31A1	**0.098873**	0.001053**
**AC132807.2**	SLC31A1	**0.083058**	0.005958**
AC129926.2	SLC31A1	0.026766	0.376244

*P-value < 0.05; **P-value < 0.01; ***P-value < 0.001; values in bold indicate that these results are statistically significant.

### Role of SLC31A1 in chemosensitivity and immune response

**Figure 8:**
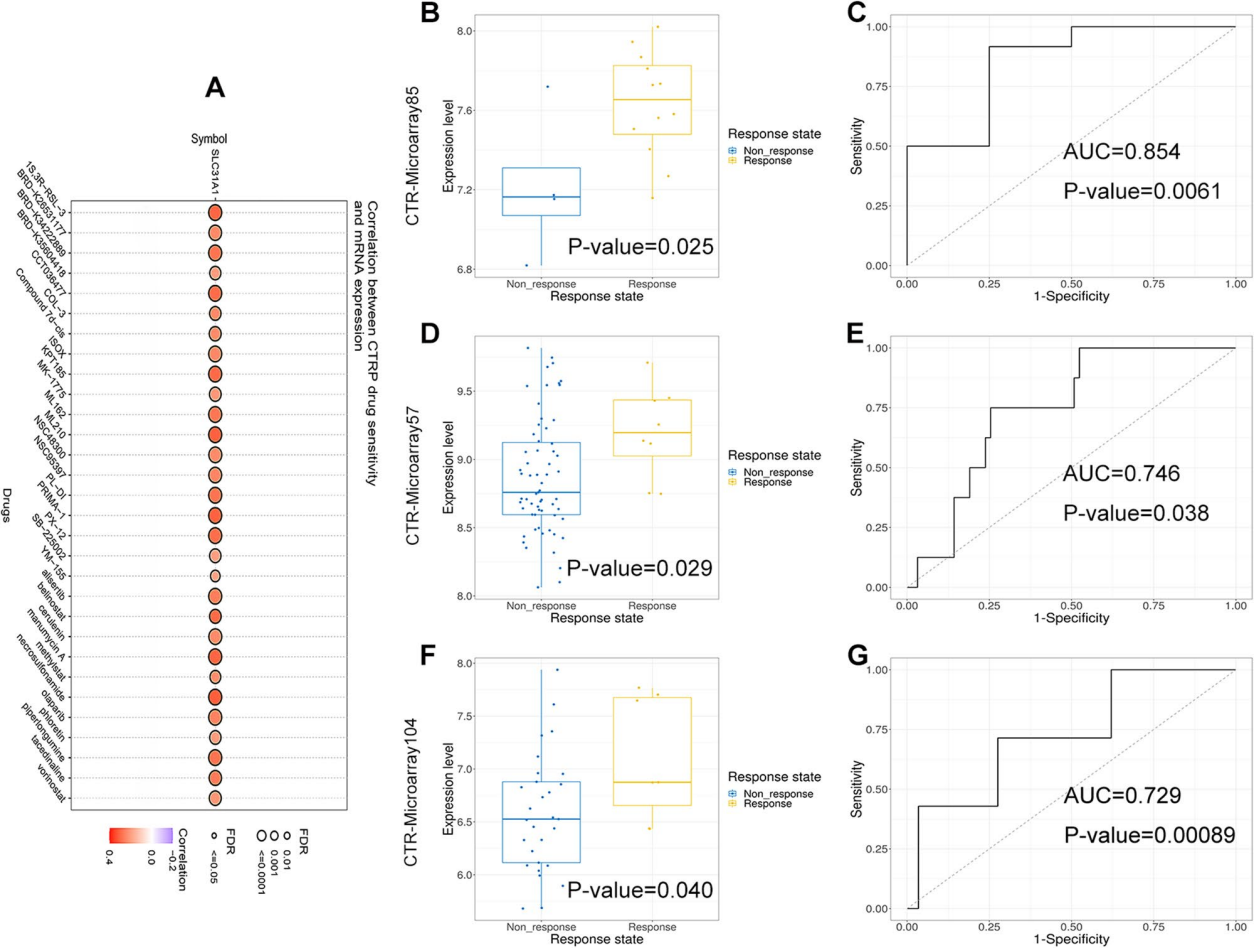
Role of SLC31A1 in chemosensitivity. (**A**) Chemosensitivity of multiple drugs regulated by SLC31A1 was predicted by GSCA. SLC31A1 expression (**B**) and AUC value (**C**) in patients treated with FEC plus docetaxel regimen was evaluated by CTR-DB. SLC31A1 expression (**D**) and AUC value (**E**) in patients treated with TA regimen was evaluated by CTR-DB. SLC31A1 expression (**F**) and AUC value (**G**) in patients treated with FEC plus paclitaxel regimen was evaluated by CTR-DB.

Since cuproptosis-related gene SLC31A1 was highly expressed and served as a promising diagnostic and prognostic marker in breast cancer, it is necessary to evaluate the role of SLC31A1 in predicting chemotherapeutic response during breast cancer treatment. By using the online tool GSCA, it was found that SLC31A1 modulated the chemosensitivity of multiple drugs (Fig. 8A). In terms of breast cancer, SLC31A1 expression was markedly decreased in non-response groups compared with response groups, when patients were treated with widely used chemotherapy regimens including FEC (fluorouracil, epirubicin, cyclophosphamide) plus docetaxel (Fig. 8B), TA (taxane, anthracycline) (Fig. 8D), and FEC (fluorouracil, epirubicin, cyclophosphamide) plus paclitaxel (Fig. 8F). Moreover, AUC was 0.854 (Fig. 8C), 0.746 (Fig. 8E), and 0.729 (Fig. 8G) in the corresponding chemotherapy regimens, revealing that SLC31A1 was able to distinguish non-response groups from response groups in breast cancer.

**Figure 9:**
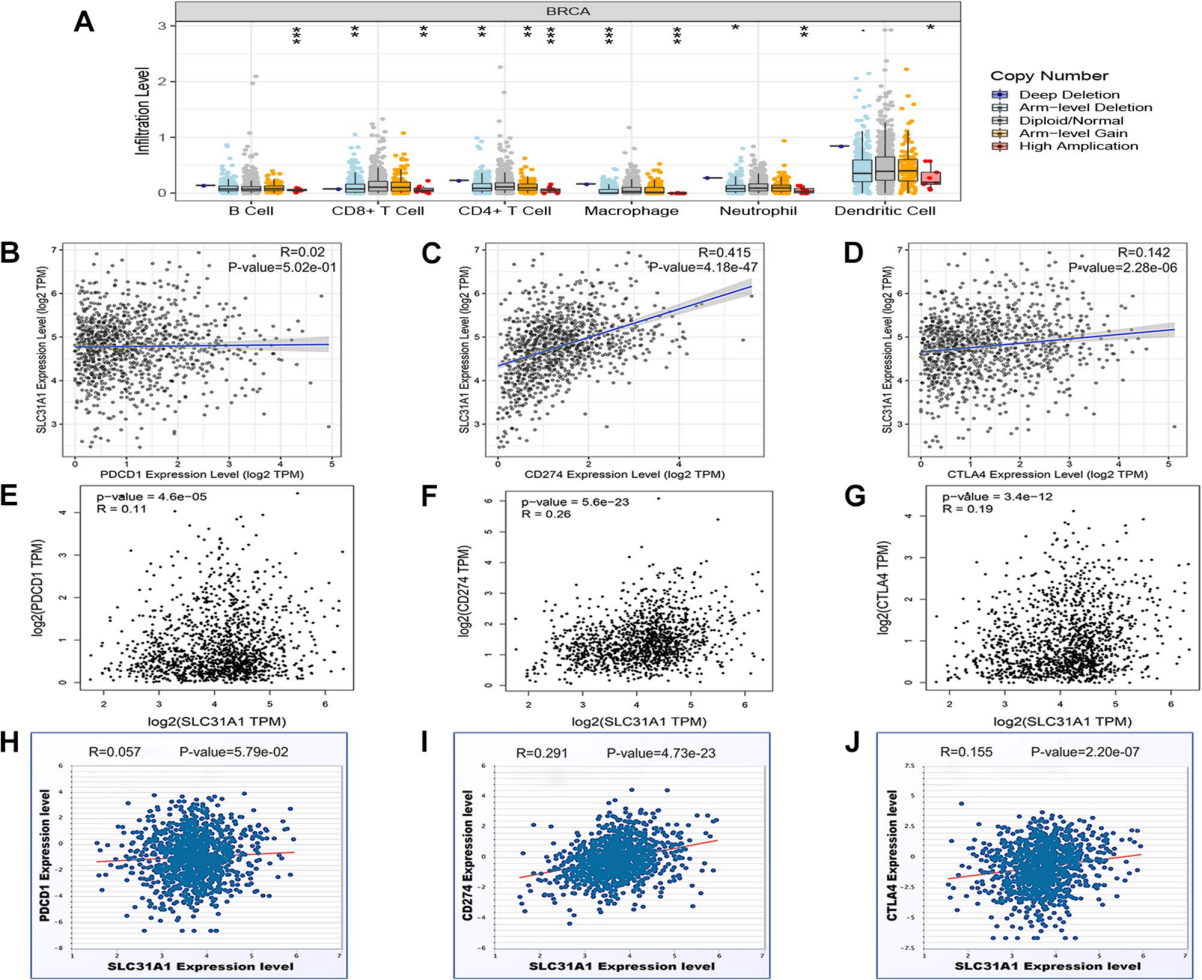
Role of SLC31A1 in immune response. (**A**) Levels of infiltrated immune cells under various copy number of SLC31A1 in breast cancer were analyzed by TIMER. Correlation analysis of SLC31A1 and immune checkpoints PDCD1 (**B**), CD274 (**C**), and CTLA4 (**D**) in breast cancer were calculated by TIMER. Correlation analysis of SLC31A1 and immune checkpoints PDCD1 (**E**), CD274 (**F**), and CTLA4 (**G**) in breast cancer were determined by GEPIA. Correlation analysis of SLC31A1 and immune checkpoints PDCD1 (**H**), CD274 (**I**), and CTLA4 (**J**) in breast cancer were analyzed by starBase.

Role of SLC31A1 in immune response was also determined. Interestingly, levels of infiltrated immune cells gradually elevated along with the increase of SLC31A1 copy number (Fig. 9A). In particular, SLC31A1 was significantly associated with infiltration degree of CD4^+^ T cells, macrophages, neutrophils, and dendritic cells. Besides, correlation analysis of SLC31A1 and immune cells biomarkers indicated that SLC31A1 was positively correlated with various immune cells biomarkers (Table 5). Given that immune checkpoints are closely related to immune response, the relationship between SLC31A1 and several immune checkpoints including PDCD1, CD274, and CTLA4 were therefore analyzed. Results from TIMER (Fig. 9B–D), GEPIA (Fig. 9E–G), and starBase (Fig. 9H–J) showed that CD274 and CTLA4, but not PDCD1, were significantly positively correlated with SLC31A1 expression. Consequently, cuproptosis-related gene SLC31A1 showed a vital role in predicting chemosensitivity and immune response.

**Table 5:** Correlation between SLC31A1 and immune cells biomarkers in breast cancer determined by GEPIA database.

Immune cells	Biomarkers	P-value	R-value
B cells	CD19	5.3E**−**02	0.052
	CD22	1E−04***	0.1
	CD79A	2.5E−07***	0.14
CD4^+^ T cells	CD4	9E−44***	0.36
CD8^+^ T cells	CD8	1.3E−19***	0.24
M1 macrophages	CD80	9.5E−79***	0.45
	CD86	4.2E−71***	0.45
	NOS2	2.4E−03**	0.082
M2 macrophages	CD115	7E−20***	0.24
	CD163	6.6E**−**01	0.012
	CD206	8.3E**−**01	0.0057
	PDL2	6.6E−06***	0.12
NK	NCR1	1.1E−04***	0.1
	SLAMF7	6.9E−40***	0.35
Fibroblasts	CD47	1.2E−50***	0.39
	ITGB1	1E−71***	0.46
Dendritic cells	ITGAX	5.4E−10***	0.17
Neutrophils	CCR7	1E−04***	0.1
	ITGAM	8.6E−26***	0.28

*P-value < 0.05; **P-value < 0.01; ***P-value < 0.001; values in bold indicate that these results are statistically significant.

## Discussion

Breast cancer is the most common cancer among women worldwide^1^. Precise surgery, adjuvant chemotherapy, and even immune therapy have greatly improved the overall survival, but prognosis of breast cancer patients remains unsatisfied^2^. Current anti-cancer therapy is based on clinical, pathological, and molecular features, and a novel approach to predict diagnosis, prognosis, and chemosensitivity is urgently required. Therefore, discovering some more promising and effective biomarkers and a significant signaling axis is of crucial importance.

Cuproptosis is a novel form of programmed cell death triggered by accumulation of intracellular copper level and aggregation of mitochondrial lipoylated proteins^3^. Mounting evidence indicate that cuproptosis contributes to cancer development and progression^4^. However, a comprehensive analysis regarding the expression patterns, diagnostic and prognostic values, and regulatory network of cuproptosis-related genes is still lacking and need to be deeply investigated. By using R language and multiple online databases on 17 candidate cuproptosis-related genes, CDKN2A and SLC31A1 were found to be increased in breast cancer samples with respect to normal tissues. After analyzing and confirming their prognostic values in breast cancer, SLC31A1 was selected as the most potential gene responsible for cuproptosis. SLC31A1 is a trans membrane protein maintaining copper homeostasis that has been recently reported to negatively contribute to cisplatin resistance in various cancers including osteosarcoma and epithelial ovarian cancer^25,26^. In the present study, SLC31A1 expression was significantly decreased in non-response groups with respect to response groups, when breast cancer patients were treated with conventional chemotherapy regimens including FEC plus docetaxel, TA, and FEC plus paclitaxel. Opposite to the above results, Takeda et al.^27^ reported that SLC31A1 was predominantly expressed in triple-negative breast cancer resistant to TA-based neoadjuvant chemotherapy. Thus, there is a major need to evaluate SLC31A1 expression in different molecular subtypes of breast cancer treated with different chemotherapy regimens.

Research over the past few years has indicated that miRNAs are a class of single-stranded and noncoding RNA with a capacity for posttranscriptional regulation of gene expression^24^. In the present work, upstream miRNAs of SLC31A1 were comprehensively predicted, after which Cox regression analysis, correlation calculation, ROC curve construction, and survival evaluation were performed. Among the candidate miRNAs, miR-204-5p was considered as the most potential upstream miRNA of cuproptosis-related gene SLC31A1. Several publications have shown that miR-204-5p acts as a tumor suppressor in many types of cancer including colorectal cancer, glioma, and thyroid carcinoma^28–30^. However, in breast cancer, studies on miR-204-5p expression show conflicting results, suggesting a possible dual regulatory role. While some investigations supported a tumor suppressor role of miR-204-5p in regulating proliferation, metastasis, and immune microenvironment remodeling in basal-like breast cancer cells^31^, other studies showed up-regulation of miR-204-5p in breast cancer tissues and the pro-proliferative effect of miR-204-5p in luminal breast cancer cells^32^. Given that no relevant reports on miR-204-5p and SLC31A1 are currently available, future studies investigating the regulation of miR-204-5p in SLC31A1 are needed in different breast cancer subtypes with distinctly different biology.

In recent years, lncRNAs have been reported to take part in various biological processes via regulating multiple molecules including miRNAs^33^. In the present work, LINC01614 was identified as the potential upstream lncRNA of miR-204-5p, after a series of lncRNAs prediction, Cox regression analysis, correlation calculation, and survival evaluation. Previous studies have showed that LINC01614 functioned as an oncogenic lncRNA in several malignancies, such as pancreatic cancer, papillary thyroid carcinoma, and gastric cancer^34–36^. In breast cancer, lncRNA transcriptional landscape identified LINC01614 as a non-favorable prognostic biomarker. Highest expression of LINC01614 was observed in luminal and HER2 + breast cancer subtypes, while lowest expression was in basal-like breast cancer subtype^37^. Moreover, LINC01614 was found to regulate epithelial-mesenchymal transition and tamoxifen sensitivity in luminal breast cancer cells^38^. Up to now, there is no direct evidence reporting the regulation of LINC01614 on miR-204-5p. It is therefore desired that further attention be drawn to this field.

Tumor microenvironment, composed of cancer cells, surrounding fibroblasts, and tumor-infiltrating immune cells, play an essential role in oncogenesis and immunotherapy efficacy^39^. In the present study, SLC31A1 was significantly associated with infiltration degree of immune cells and levels of immune cells biomarkers. In addition, SLC31A1 was positively related to the expressions of immune checkpoints CD274 and CTLA4, suggesting that targeting SLC31A1 might be a potential way to improve immunotherapy efficacy in breast cancer.

The present study has some significant advantages. First, while most of the publications reporting SLC31A1 were based on limited data derived from online tools, the current work utilized R language to obtain TCGA breast cancer information in a more synchronous and comprehensive manner. This approach offered more rigorous analysis and more convincing results. Second, the present study is the first to propose that LINC01640/miR-204-5p/SLC31A1 might be a significant and promising axis during cuproptosis in breast cancer, whereas other bioinformatics analysis only focused on the expression levels, prognostic and diagnostic values, and immune regulation of cuproptosis-related gene SLC31A1. There are also certain limitations to be thoroughly considered. On one hand, the detailed molecular subtypes and treatment information were not available for data analysis, which might have potential influence on the results; on the other hand, statistical analysis was based on data derived from public-available databases, and further biological experiments and clinical verification are required in the future.

## Conclusion

In summary, this study found that SLC31A1 was a potential cuproptosis-related gene, which was significantly upregulated and was able to predict diagnosis, prognosis, chemosensitivity, and immune infiltration in breast cancer. LINC01640/miR-204-5p/SLC31A1 might be a significant and promising axis during cuproptosis in breast cancer.

### Supplementary Information

Supplementary Figure 1.

Supplementary Table 1.

## Data Availability

The data sets analyzed during the current study are available from the corresponding author on reasonable request.
