# Supplementary material for: Identification of cuproptosis-related gene SLC31A1 and upstream LncRNA-miRNA regulatory axis in breast cancer
Source: Sci Rep. 2023 Oct 26;13:18390. doi: 10.1038/s41598-023-45761-5 (PMC10603161; doi:10.1038/s41598-023-45761-5)
Supplement: Supplementary file 1 — Supplementary Figure 1. [file 41598_2023_45761_MOESM1_ESM.pdf]

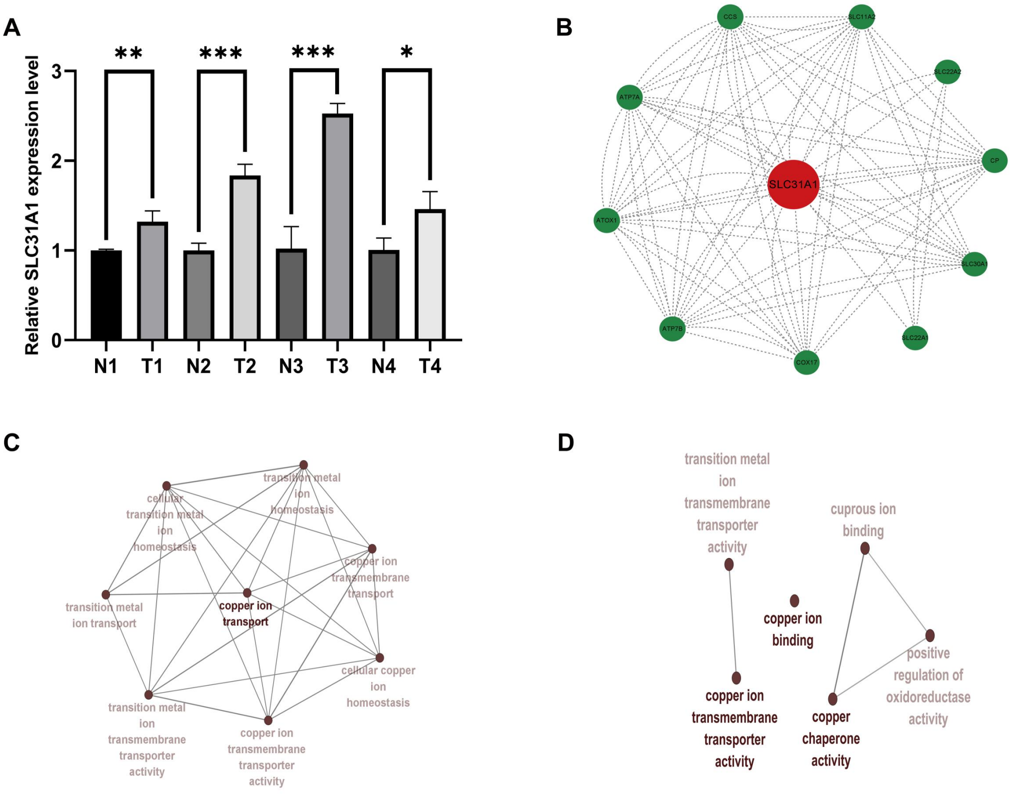

Supplementary Figure 1. Expression, PPI network, and enrichment analysis of SLC31A1 in breast cancer. (A) Expression level of SLC31A1 in 4 pairs of breast cancer samples and adjacent normal breast tissues obtained from own hospital. * $P < 0.05$, **$P < 0.01$, *** $P < 0.001$. (B) Analysis of PPI network. (C) Enrichment analysis of biological process of SLC31A1-interacting proteins. (D) Enrichment analysis of molecular function of SLC31A1-interacting proteins.
