# Supplementary material for: Identification of cuproptosis-related gene SLC31A1 and upstream LncRNA-miRNA regulatory axis in breast cancer
Source: Sci Rep. 2023 Oct 26;13:18390. doi: 10.1038/s41598-023-45761-5 (PMC10603161; doi:10.1038/s41598-023-45761-5)
Supplement: Supplementary file 2 — Supplementary Table 1. [file 41598_2023_45761_MOESM2_ESM.docx]

**Supplementary table 1: Primer sequences used for PCR.**

| **Gene** | **Primer** | **Sequence (5**' **→ 3**'**)** |
| --- | --- | --- |
| SLC31A1 | Forward primer | TGATGCCTATGACCTTCTACTTTG |
|  | Reverse primer | TCGGGCTATCTTGAGTCCTTC |
| β-actin | Forward primer | TCAAGATCATTGCTCCTCCTGAG |
|  | Reverse primer | ACATCTGCTGGAAGGTGGACA |
